# Psychometric properties of screening tools for mild cognitive impairment in older adults based on COSMIN guidelines: a systematic review

**DOI:** 10.1186/s12877-025-06030-4

**Published:** 2025-06-02

**Authors:** Shasha Wen, Dongmei Cheng, Nana Zhao, Xinyu Chen, Xianying Lu, Yue Li, Huanle Liu, Jing Gao, Chaoming Hou, Ran Xu

**Affiliations:** 1 School of Nursing, Chengdu University of Traditional Chinese Medicine, Chengdu, Sichuan Province China; 2 Chengdu University of Traditional Chinese Medicine, Chengdu, Sichuan Province China; 3 Chengdu Third People‘s Hospital, Chengdu, Sichuan Province China

**Keywords:** Instruments, Psychometric properties, Systematic evaluation, Mild cognitive impairment

## Abstract

**Background:**

The prevalence of mild cognitive impairment in older adults is understood to be as high as 40%, and early screening for MCI may slow the progression of Alzheimer's disease. However, no systematic review has summarized the psychometric properties of instruments.

**Objective:**

This systematic review aimed to assess the psychometric properties of existing scales for screening older adults for mild cognitive impairment and to provide an evidence-based basis for selecting the most appropriate assessment tool for older adults.

**Design:**

This study systematically reviewed the measurement properties using the consensus-based Criteria for the Selection of Instruments for Measuring Health (COSMIN) method.

**Methods:**

Eight electronic databases (PubMed, Embase, Web of Science, Scopus, Cochrane, CNKI, Wanfang, and Proquest) were systematically searched from inception up to October 26, 2024. Methodological quality was assessed using the COSMIN risk of bias checklist, and psychometric properties were summarized and evaluated using the COSMIN criteria.

**Results:**

Thirty-one studies reported 30 different versions of screening instruments, with 15 studies examining more than 5 psychometric properties. Limited information on construct validity and reliability was found. No data were found on cross-cultural validity/measurement invariance, measurement error, or responsiveness. The final three instruments, AV-MoCA, HKBC, and Qmci-G, received class A recommendations and were recommended for use. The TICS-M study had insufficient psychometric properties and received a class C recommendation; thus, it was not recommended for use. The other 26 instruments were class B recommendations, indicating potential for use, although further research is needed to assess their psychometric properties.

**Conclusion:**

The AV-MoCA, HKBC, and Qmci-G can be used to screen older adults for MCI. Future research is needed to further validate the cross-cultural applicability of these instruments and to fully assess their psychometric properties.

**Supplementary Information:**

The online version contains supplementary material available at 10.1186/s12877-025-06030-4.

## Background

In the context of a globally aging population, the health of older adults has emerged as a major concern According to the World Population Prospects 2022 report by the United Nations Population Division (UNPD), individuals aged 60 years and over constitute 13.7% of the total population [1]. Projections indicate that this demographic is expected to reach 2.1 billion by 2050 [2]. Mild cognitive impairment (MCI) is a prevalent age-related health problem that represents an intermediate state between typical age-related cognitive changes and early dementia [3]. MCI typically leads to cognitive decline, which affects the ability to perform daily activities [4], and is associated with increased levels of anxiety and depression, which can interfere with daily life and well-being [5]. The prevalence of MCI in older adults ranges from 28 to 40% [6], with an estimated one-third of people over the age of 65 experiencing MCI. Current evidence suggests that the incidence of MCI in the 75–79 age group is 22.5/1000 people per year, while the prevalence rises to 60% in the 85 and older age group, posing a significant public health challenge [7, 8]. Notably, people with MCI are at increased risk of potential dementia, with approximately 10–15% of people with MCI developing dementia, placing a significant burden on patients, caregivers, families, and society [9].

Early identification and intervention for individuals with MCI are imperative to counter disease progression, enhance patient quality of life, and mitigate healthcare burden [10–12]. Research has demonstrated that individuals with MCI exhibit a tenfold increased likelihood of developing dementia compared to those who are cognitively healthy [13]. A meta-analysis of 12,000 patients also showed that patients with amnestic MCI had a 60% chance of progressing to AD within 5 years without timely intervention, whereas early intervention reduced the risk of conversion by 35–50% [4], demonstrating the importance of early detection and intervention to prevent or delay progression to dementia in patients with MCI [14]. Consequently, screening and early diagnosis of mild cognitive impairment in high-risk populations are imperative [15, 16].

The diagnosis of MCI relies on a comprehensive neuropsychological assessment, with the caveat that a mere five-minute evaluation has been shown to enhance clinicians'MCI recognition rates threefold [17]. However, numerous disparate measurement tools are currently employed for MCI screening in older adults, exhibiting a spectrum of psychometric properties [18, 19]. Psychometric properties, which are crucial for evaluating measurement tools, encompass content validity, structural validity, internal consistency, and reliability. These properties directly impact the accuracy and reliability of screening results, which in turn influence clinical decision-making and patient management (Peng et al., 2024). The utilization of assessment tools lacking reliable psychometric characteristics can compromise the accuracy of study findings, thereby augmenting the risk of misdiagnosis and underdiagnosis during the screening of older adults with MCI.

In recent years, scholars have explored the importance of screening tools to detect MCI. However, most of these studies have not employed standardized methodologies to assess the psychometric properties of the tools, resulting in limited scientific validity and reliability of the conclusions [20, 21]. For example, Abd Razak et al. (2019) evaluated the sensitivity and specificity of screening tools for patients with MCI and AD, without examining other crucial psychometric properties. Peng et al. (2024) comprehensively reviewed 156 studies of 19 instruments; however, the application of these instruments'measures to an overly broad population and the lack of specific screening tools for older patients with MCI complicate the selection of appropriate measurement tools for this population to ensure the scientific rigor and validity in screening. The COnsensus-based Standards for the selection of health Measurement INstruments (COSMIN) initiative refers to a set of international consensus-based standardized instruments designed to systematically assess the psychometric properties of health measurement instruments and to promote the standardization of health outcome measures and the standardization of instrument selection [22]. The 2021 update of the COSMIN Risk of Bias Inventory expanded [23] its framework for assessing clinician-reported outcomes (ClinPOMs) and performance-based outcome measures (PerFOMs). Based on the guidelines published in 2018, this version expands the risk of bias assessment criteria for reliability and measurement error, with emphasis on the characteristics of ClinROMs and PerFOMs measurement tools that require professional personnel to operate the equipment or instruct patients. Currently, this inventory is widely used in clinical research, as exemplified by COSMIN-standardized assessment tools for conditions such as delirium [24], frailty [25], and developmental central hypotonia [26]. In our study, the cognitive screening tools examined for older patients with MCI fell into the ClinPOMs category. Accordingly, we aimed to utilize the COSMIN criteria to systematically evaluate the psychometric properties of measurement tools for screening older adults with MCI. By summarizing critical expert and patient metrics regarding the measurement properties of these tools, we comprehensively assessed and compared these properties to provide an evidence-based basis for selecting appropriate screening tools. Moreover, future research directions were identified to promote the scientific and effective development of screening for MCI in older adults.

## Methodology

This systematic review was conducted in accordance with the COSMIN methodology for systematic reviews of psychometric properties [27–29] and the Reporting Items for Systematic Reviews and Meta-Analyses (PRISMA) statement [30] (See supplementary appendix A). We prospectively registered the current review in the International Prospective Register of Systematic Reviews (PROSPERO) database (registration number: CRD 42024605647).

### Search strategy

The search strategy employed in this study comprised three steps. Initially, an initial search was conducted in PubMed using MeSH terms and free terms to develop search terms. This identified search strategy was then confirmed by the research team. Subsequently, a literature search was conducted in eight databases, including PubMed, Embase, Web of Science, Scopus, Cochrane, CNKI, Wanfang, and the grey literature database Proquest, employing the aforementioned search strategy. The search was conducted from the inception up to October 26, 2024, and the COSMIN filter was applied to the feasible databases [31]. The references of all included studies were reviewed to supplement the eligible literature that was not included in the search strategy. The search strategies employed for each database are detailed in Appendix 1.

### Inclusion and exclusion criteria

The inclusion criteria were as follows: 1. Studies targeting older adults (≥ 60 years old) (According to the United Nations'definition, people aged 60 years and older are categorized as older adults [32]); 2. Studies that aimed to develop or validate an outcome measurement instrument reported by older adults with mild cognitive impairment and assessed one or more psychometric properties; and 3. Studies published in English or Chinese. The definition of mild cognitive impairment in our study followed the revised 2003 International Working Group diagnostic criteria for MCI [33].

The exclusion criteria comprised the following: 1. Studies for which the full text was not available; 2. Studies that were duplicates or overlapped with other publications; 3. Studies that provided indirect evidence of psychometric properties; 4. Secondary literature, such as reviews, systematic evaluations, or meta-analyses.

### Study screening and selection

The initial step in the selection process involved importing all references retrieved from relevant databases, followed by the removal of duplicates using EndNote 21. Two researchers (SSW and DMC), trained in evidence-based methods, independently screened the references. The screening process commenced with a preliminary examination of titles and abstracts, and was followed by a comprehensive review of the full text. This process was undertaken to identify studies that met the predetermined inclusion and exclusion criteria, with each exclusion being meticulously documented. A third researcher (NNZ) was tasked with resolving any disagreements that arose throughout the study selection process.

### Data extraction

The data extraction process involved the retrieval of information from the included studies and the characteristics of patient-reported outcome measures. As illustrated in Table 1, the study characteristics encompassed the authors (year), the country or region where the study was conducted, the language of the patient-reported outcome measures, the study design, the sample size, the mean education level of older adults with MCI, and the mean age of older adults with MCI. Table 2 presents the characteristics of patient-reported outcome measures, including references, target population, mode of administration (self-reported, interview-based), number of subscales and entries, score ranges, time to scale completion, time to retest, and diagnostic cutoff scores. Two researchers (SSW and DMC) independently extracted data and information using Tables 1 and 2, respectively. A third researcher (NNZ) was invited to discuss any inconsistencies and disagreements.

**Table 1:** Study characteristics

Authors (Year)	Study design	Patient-reported utcome measure	Patient-reported utcome measure shortened form	Country/region	Original language/translation	Sample size	MCI average education years	MCI average age
Bentvelzen et al. (2019) [34]	Longitudinal	Modified Telephone Interview for Cognitive Status	TICS-M	Australia	English	617	11.7 ± 11.6	79.7 ± 4.7
Broche-Pérez and López-Pujol (2017) [35]	Cross sectional	Cuban Version of Addenbrooke’s Cognitive Examination—Revised	ACE-R	Cuba	English/Spanish	129	8.6 ± 3.7	75.4 ± 7.9
Calderón et al. (2021) [36]	Cross sectional	Addenbrooke’s Cognitive Examination III -Chilean version	ACE-III-S	Chile	English/Spanish	1164	NR	71.8 ± 7.9
Carvalho et al. (2024) [37]	Cross sectional	Audiovisual version of the Montreal Cognitive Assessment (MoCA)	AV-MoCA	Brazilian	English/Brazilian Portuguese	114	15.4 ± 3.9	69.0 ± 6.8
Chiu et al. (2018) [38]	Cross sectional	Hong Kong Brief Cognitive Test	HKBC	China	Chinese	359	5.6 ± 4.6	76.4 ± 6.8
Delgado et al. (2019) [39]	Cross sectional	Montreal Cognitive Assessment en Latinoamérica	MoCA-S	Chile	English/Spanish	172	11.0 ± 4.0	73.0 ± 6.0
Erdoğan et al. (2024) [40]	Cross sectional	Rapid Cognitive Screen—Turkish version	RCS—TR	Turkey	English/Turkish	172	NR	75.4 ± 8.5
Freitas et al. (2013) [41]	Cross sectional	Montreal Cognitive Assessment (MoCA)	MoCA -P	Portugal	English/Portugal	360	6.5 ± 4.5	70.5 ± 8.0
Fujiwara et al. (2010) [42]	Cross sectional	Japanese version of the Montreal Cognitive Assessment	MoCA—J	Japan	English/Japanese	96	11.5 ± 3.1	77.3 ± 6.3
Freedman et al. (2018) [43]	Cross sectional	Toronto Cognitive Assessment-iPad version	TorC-iPad version	Canada	English	107	15.5 ± 3.4	77.7 ± 6.5
Girtler et al. (2012) [44]	Cross sectional	Short Cognitive Evaluation Battery Italian version	SCEB-I	Italy	French/Italian	131	7.3 ± 4.3	76.6 ± 6.5
Hamilton et al. (2022) [45]	Longitudinal	Composite Autonomic Symptom Score 31-item scale	COMPASS	USA	English	126	NR	74.7 ± 7.5
Jeong et al. (2004) [46]	Cross sectional	Korean version of modified Mini-Mental State Examination	K-mMMSE	South Korea	English/Korean	522	NR	73.5 ± 6.7
Koc Okudur et al. (2019) [47]	Longitudinal	Turkish version of the Rapid Cognitive Screen	RCS-T	Turkey	English/Turkish	323	8.47 ± 3.9	72.2 ± 7.4
Lee et al. (2008) [48]	Cross sectional	Korean version of the Montreal Cognitive Assessment	MoCA—K	South Korea	English/Korean	196	8.3 ± 3.8	71.3 ± 5.9
Lee et al. (2018) [19]	Cross sectional	Taiwan version of the Quick Mild Cognitive Impairment screen	Qmci-TW	China	English/Chinese	102	7.3 ± 4.9	77.1 ± 7.5
Manser and de Bruin (2024) [49]	Cross sectional	Quick mild cognitive impairment screen	Qmci-G	Switzerland	English/German	80	14.9 ± 4.0	77.0 ± 10.0
Memõria et al. (2013) [50]	Cross sectional	Brazilian Version of the Montreal Cognitive Assessment	MoCA—BR	Brazil	English/Brazilian Portuguese	112	11.41 ± 4.23	74.30 ± 5.60
Morita et al. (2019) [51]	Cross sectional	Japanese version of the Quick Mild Cognitive Impairment	Qmci-J	Japan	English/Japanese	526	NR	73.5 ± 5.6
Muñoz‐Neira et al. (2014) [52]	Cross sectional	Test Your Memory—Spanish version	TYM-S	Chile	English/Spanish	74	NR	NR
Nasreddine et al. (2005) [53]	Cross sectional	Montreal Cognitive Assessment (MoCA)	MoCA	Canada	English	277	12.0 ± 4.32	75.19 ± 6.27
Potts et al. (2022) [54]	Cross sectional	Addenbrooke’s Cognitive Examination III	ACE-III	Northern Ireland	English	2,176	NR	79.6 ± 7.5
Rami et al. (2007) [55]	Cross sectional	Memory Alteration Test	M@T	Spain	Spanish	610	8.4 ± 5.2	76.6 ± 6.6
Razali et al. (2014) [56]	Cross sectional	Montreal Cognitive Assessment—Bahasa Malaysia version	MoCA—BM	Malaysia	Malaysia	180	NR	65.3 ± 5.4
Rashedi et al. (2019) [57]	Cross sectional	Persian version of general practitioner assessment of cognition	PGPCOG	Iran	English/Persian	230	NR	70.7 ± 9.5
Sala et al. (2020) [58]	Cross sectional	Japanese version of the Montreal Cognitive Assessment	MoCA—J	Japan	English/Japanese	2408	NR	NR
Špeh et al. (2024) [59]	Cross sectional	Slovenian version of the Montreal Cognitive + D3:D35 Assessment Scale	MoCA -S	Slovenian	English/Slovenian	93	11.8 ± 3.3	74 ± 6.6
Vanoh et al. (2016) [60]	Cross sectional	TUA-WELLNESS	TUA-WELLNESS	Malaysia	Malay	1,993	NR	68.5 ± 5.93
Xue et al. (2018) [61]	Cross sectional	Six-Item Screener	SIS	China	English/Chinese	373	NR	71.6 ± 8.7
Yu et al. (2012) [62]	Cross sectional	Beijing version of the Montreal Cognitive Assessment	MoCA -BJ	China	English/Chinese	1001	8.43 ± 5.46	71.45 ± 7.26
Yun Sun (2022) [63]	Cohort	Addenbrooke’s Cognitive Examination III -China version	ACE-III-C	China	English/Chinese	104	7.7 ± 1.1	70.1 ± 3.3

*NR* Not reported

**Table 2:** Patient-reported outcome measure characteristics

PROM	Target population	Mode of administration	Dimensions	Subtypes	Number of items	Range of scores	Scale completion time(minutes)	Recall period	Diagnostic cut-offs
TICS-M [34]	Older adults	Interview-based telephone	5	Orientation, memory, abstract thinking, language, attention	13	0–39	5–10	NR	≤ 24
ACE-R [35]	Older adults aged 60 and above	Interview-based	5	Attention/orientation, memory, verbal fluency, language, visuospatial functions	81	0–100	15	NR	≤ 84
ACE-III-S [36]	Older adults aged 60 and above	Interview-based	5	Attention, memory, fluency, language, visuospatial ability	81	0–100	NR	NR	86
AV-MoCA [37]	Older adults aged 60 and above	Interview-based email or by phone call	6	Attention, executive functions, visuospatial abilities, language, memory, orientation	13	0–30	NR	NR	≥ 23
HKBC [38]	Older adults aged 65 and above	Interview-based	5	Immediate recall/attention, delayed recall, recent memory, orientation, frontal lobe function test, general knowledge, visuospatial construction, executive function, and language	9	0–30	7	4 weeks	21/22
MoCA-S [59]	Older adults aged 60 and above	Interview-based	6	Attention, concentration, executive functions, memory, language, visuospatial skills, abstraction, calculation, orientation	30	0–30	10–15	NR	23/24
RCS – TR [40]	Older adults aged 60 and above	Interview-based	3	Recall of five words, a clock drawing test, and the ability to remember a story and convert the facts	3	0–10	3	2 weeks	≤ 8
MoCA -P [41]	Older adults aged 60 and above	Interview-based	8	Executive functions; visuospatial abilities; short—term memory; language; attention, concentration and working memory; and temporal and spatial orientation	30	0–30	10–15	3 months	< 22
MoCA – J [42]	Older adults	Interview-based	8	Visuospatial/executive function, naming, attention, language, memory, abstraction, calculation and orientation	30	0–30	10	8 weeks	25/26
TorC-iPad version [43]	Older adults aged 60 and above	Interview-based	7	Orientation, Immediate Recall, Delayed Recall, Delayed Recognition, Visuospatial Function, Working Memory/Attention/Executive Control, and Language	27	No upper limit	30	28–120 days	275
SCEB-I [44]	Older adults aged 65 and above	Interview-based	4	Temporal orientation test, 5-word test, clock drawing test, semantic verbal fluency test	13	0–36	6–12	NR	1
COMPASS [45]	Older adults aged 60 and above	Self-report	6	Autonomic dysfunction; orthostatic intolerance, vasomotor, secretomotor, Pupillomotor, gastrointestinal and bladder symptoms	31	0—40	NR	NR	4/5
K-mMMSE [46]	Older adults aged 65 and above	Interview-based	4	Political figures, word fluency, similarities, and delayed recall	11	0–100	NR	26 days	69/70
RCS-T [47]	Older adults aged 60 and above	Interview-based	3	Recall of 5 words, clock drawing test, remember a story	3	0–10	NR	NR	≤ 6
MoCA-K [48]	Older adults	Interview-based	6	Visuospatial/executive function, naming, attention, language, memory, and orientation	30	0–30	NR	4 weeks	22/23
Qmci-TW [19]	Older adults aged 65 and above	Interview-based	6	Orientation, registration, clock drawing, delayed recall, verbal fluency, and logical memory	6	0–100	5	2 weeks	≤ 51.5
Qmci-G [49]	Older adults aged 60 and above	Self-report	6	Orientation, registration, clock drawing delayed recall, verbal fluency and logical memory	6	0–100	5	NR	≤ 67
MoCA – BR [50]	Older adults aged 65 and above	Interview-based	6	Short—term memory, visuospacial abilities, executive function, attention/concentration/working memory, language, orientation	30	0–30	NR	3 months	25
Qmci-J [51]	Older adults aged 65 and above	Interview-based	6	Orientation, word registration, clock drawing, delayed recall, verbal fluency, logical memory	6	0–100	6	NR	56/57
TYM-S [52]	Older adults aged 65 and above	Self-report	10	Orientation, copying, semantic knowledge, calculation, verbal fluency, abstraction, naming, visuospatial abilities, anterograde memory, executive function	10	1—50	NR	NR	44
MoCA [53]	Older adults aged 60 and above	Interview-based	8	Executive functions; visuospatial abilities; short—term memory; language; attention, concentration and working memory; and temporal and spatial orientation	30	0–30	10	3 months	< 26
ACE-III [54]	Older adults aged 65 and above	Interview-based	5	Attention, memory, language fluency, language, visuospatial ability	81	0–100	NR	NR	84
M@T [55]	Older adults aged 60 and above	Interview-based	2	Animal Fluency Test, Delayed Word List Recall	11	0–30	15	NR	15/16
MoCA – BM [56]	Older adults aged 60 and above	Interview-based	8	Attention and concentration, executive function, memory, language, visuoconstructional skills, conceptual thinking, calculations and orientation	30	0–30	10–15	NR	22/23
PGPCOG [57]	Older adults aged 60 and above	Interview-based	2	Cognitive and informant	15	0–15	NR	19 days	≤ 6
MoCA – J [58]	Older adults	Interview-based	8	Visuospatial/executive function, naming, attention, language, memory, abstraction, calculation and orientation	30	0–30	10	NR	NR
MoCA-S [39]	Older adults aged 60 and above	Interview-based	6	Visuoespatial/executive, identification, attention, language, abstraction, delayed recall, and orientation	30	0—30	10	10 days	≤ 21
TUA-WELLNESS [60]	Older adults aged 60 and above	Self-report	10	Sociodemographics, comorbidities, fitness, nutritional and functional status, Psychosocial, dietary intake and practice, lifestyle	10	0–17	NR	NR	≥ 11
SIS [61]	Older adults aged 60 and above	Interview-based	3	Memory, Calculation, Orientation	6	0–6	1	NR	≤ 3
MocA-BJ [62]	Older adults aged 60 and above	Interview-based	7	Visuospatial/executive function, naming, attention, abstraction, language, delayed memory, and orientation	30	0–30	NR	NR	21/22
ACE-III-C [63]	Older adults aged 60 and above	Interview-based	5	Attention, memory, language fluency, language, visuospatial ability	81	0–100	15	4 weeks	82/83

*NR* Not reported

### Assessment of methodological quality

Two researchers (SSW and DMC) independently assessed the methodological quality of each study using the COSMIN risk of bias checklist [27, 29, 64]. Any inconsistencies or disagreements were resolved through mediation by a third researcher (NNZ). The COSMIN Risk of Bias Checklist comprises 10 primary items and 116 additional items, encompassing patient-reported outcome measure development, content validity, structural validity, internal consistency, cross-cultural validity/measurement invariance, reliability, measurement error, criterion validity, hypothesis testing for structural validity, and responsiveness. In addition, the 2021 updated version of the COSMIN risk of bias checklist [23] was used for the assessment of reliability and measurement error. Each item was appraised on a five-point scale, ranging from"very good"to"not applicable", with the latter indicating that the item was not applicable to the specific context under review. The"worst score count"principle was employed to determine the overall quality of the domain of interest, whereby the lowest score for all entries under an attribute determined the overall risk of bias score for that attribute.

### Summarizing the quality of psychometric properties

Two researchers (SSW and DMC) independently summarized the quality of the psychometric properties of each patient-reported outcome measure according to the COSMIN criteria [65]. A third researcher (NNZ) was invited to discuss any inconsistencies and disagreements. The COSMIN criteria rated the psychometric properties of the patient-reported outcome measures (including content validity, structural validity, internal consistency, cross-cultural validity/measurement invariance, reliability, measurement error, criterion validity, hypothesis testing for structural validity, and responsiveness) as adequate (+), indeterminate (?), or insufficient (-). Initially, the psychometric properties of each individual study were evaluated, and subsequently, a synthesis of the results was conducted to formulate a conclusive appraisal of the quality of the psychometric properties of the outcome measures in their entirety, on an individual basis. The overall ratings assigned to the pooled or aggregated results included adequate (+), inadequate (-), inconsistent (±), or indeterminate (?). In instances where the results of a study were deemed adequate (or inadequate in their entirety), the overall rating remained consistent with this evaluation. Conversely, if the results of studies that could be combined were inconsistent and the inconsistency could not be explained, the combined results were rated as adequate or inadequate, with a downgrade applied for the presence of inconsistency. During assessment of content validity, the reviewers'evaluation of the PROM itself was also considered. The evaluators'qualitative ratings were used to determine the overall content validity of the PROM, with the results summarized as adequate (+), inadequate (-), or inconsistent (±). An indeterminate overall score (?) was not applicable due to the evaluator's rating being a constant (+, -, or ±). In the event that each study was assigned a rating of adequate (+) or inadequate (-), the resulting overall rating was also determined as adequate (+) or inadequate (-). When no content validity studies were available, or when the content validity studies were of poor quality, and furthermore, when the development of the patient-reported outcomes was of poor quality, the reviewer's rating determined the overall rating. Ratings for developmental or content validity studies were disregarded. In instances where results for psychometric properties of the same instrument were inconsistent across studies, conclusions were derived based on the most consistent results and are downgraded for inconsistency [64].

### Grading the quality of evidence

Two researchers (SSW and DMC) independently assessed the certainty of the evidence according to the revised Recommendations, Assessment, Development and Evaluation Grading System [28]. A third researcher (NNZ) was invited to discuss any inconsistencies and disagreements. The quality of the evidence was determined using a modified quantitative systematic evaluation employing the GRADE method of scoring, which includes four factors: risk of bias, inconsistency, indirectness, and imprecision. Each patient-reported outcome measure's psychometric attributes were categorized as"high","moderate","low", or"very low"evidence. Specifically, assessment of content validity exclusively considered the three factors of risk of bias, inconsistency, and indirectness of the evidence, given that PROM development and content validity studies are primarily qualitative, rendering imprecision less relevant. Besides, there was a paucity of registry information on PROM development and content validity studies. Consequently, the imprecision and publication bias factors of the evidence were not considered, and COSMIN defaulted the evidence to “high” until the beginning of the evaluation grade. According to the COSMIN methodology, the included patient-reported outcome measures were recommended to be categorized into three types of recommendations. The first type is designated as Category A recommendations, characterized by content validity that is"adequate (+)"(any level of evidence) and internal consistency that is"adequate (+)"(at least low-quality evidence). Category A recommendations are endorsed for utilization. The second type is designated as Category B recommendations. Category B recommendations are tools that do not fall into categories A or C and that have potential for application but require further research to evaluate their quality. The third category of recommendation pertains to tools for which there is substantial evidence indicating that their measurement attributes are"insufficient (-)". These tools are not recommended for use.

## Results

### Literature search

**Fig. 1:**
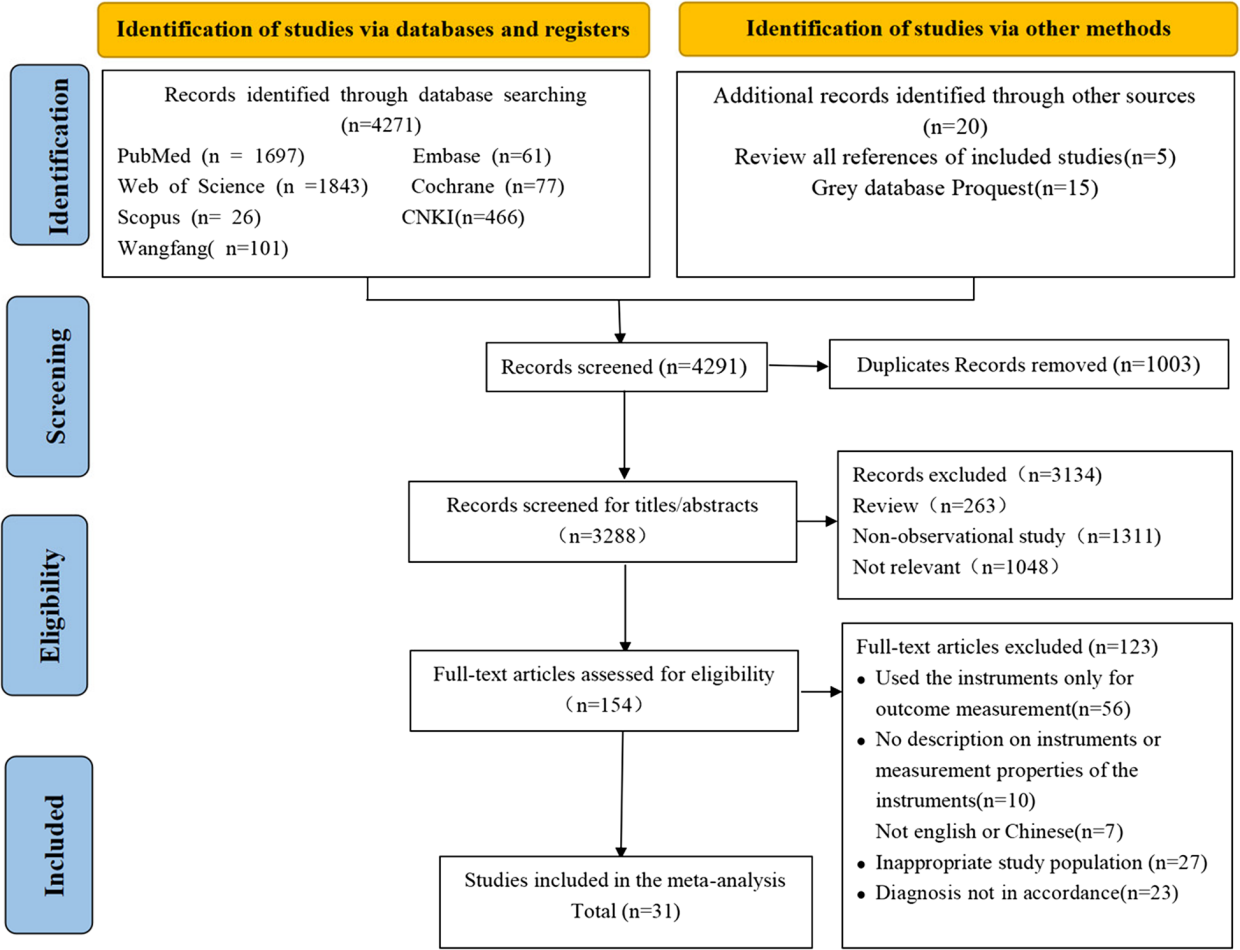
PRISMA flowchart depicting the literature screening process

A total of 4,271 studies were obtained from the initial search of seven databases. Twenty additional studies were obtained by reviewing the references of the included studies and searching the grey literature database, yielding a total of 4,291 studies. After removing 1,003 duplicates, 3,288 studies were excluded based on titles and abstracts, and 154 studies were excluded after full-text review. The final number of papers included in this study was 31, including 30 patient-reported measurement instruments. The PRISMA flow chart for the study screening process is depicted in Fig. 1.

### Description of studies and patient-reported outcome measures

Of the 31 articles included, 30 were published in English and 1 in Chinese. The publication years ranged from 2004 to 2024. Study regions included Europe, Asia, the Americas, Oceania, and Malaysia. Three longitudinal studies and one cohort study were included, alongside 27 cross-sectional studies that collectively included a total of 14,927 participants with sample sizes ranging from 74 to 2,408. The mean years of education and age of MCI patients varied across studies, with years of education ranging from 5.6 ± 4.6 to 15.5 ± 3.4, and age ranging from 65.3 ± 5.4 to 79.7 ± 4.7. The original language of most of the study scales was English, with subsequent translations into multiple languages to accommodate research requirements in different countries. The characteristics of the 31 included studies are shown in Table 1.

The patient-reported instruments encompassed various language versions, iPad versions, and audiovisual versions. The primary administration methods of the tools included face-to-face interview-based or telephone interviews (Interview-based); four tools utilized self-report, and one tool employed a combination of interview and self-report. The scales varied in the number of dimensions assessed, ranging from 2 to 8, covering attention, memory, language ability, executive function, visuospatial ability, orientation, etc. Notably, some of these scales feature subtypes, with the MoCA, for instance, presenting subtypes across different language versions that diverge in their emphasis on specific cognitive domains. The range of entries varied from 3 to 81, with scores falling within the 0 to 100 range. Most of the instruments were designed to be completed within a time frame of 5 to 15 min, a characteristic that rendered them well-suited for expeditious screening procedures. The diagnostic cutoffs exhibited variability across instruments, with certain patient-reported tools adjusting their cutoffs based on the subject's literacy level. The characteristics of the 31 patient-reported measurement tools are displayed in Table 2

### Methodological quality assessment of the included studies

COSMIN categorizes the measurement properties of scales into three aspects: validity, reliability, and responsiveness. Validity encompasses content validity, construct validity (structural validity, hypothesis testing, cross-cultural validity), and criterion validity. Reliability includes internal consistency, stability, and measurement error. Of the 31 studies that were included in the analysis, 15 studies explored more than five cardiac measurement attributes. Limited information was retrieved in terms of construct validity and reliability, and no data were found on cross-cultural validity, measurement invariance, measurement error, and responsiveness. The results of the methodological quality assessment of the included studies are presented in Table 3.

**Table 3:** Methodological quality assessment

**Patient-reported outcome measure**	**Patient-reported outcome measure development**	**Content validity**	**Structural validity**		**Internal consistency**		**Reliability**		**Criterion validity**				**Hypothesis testing for construct validity**	
	**Results of evaluation**	**Results of evaluation**	**Indicators**	**Results of evaluation**	**Cronbach's ** **a** ** coefficient**	**Results of evaluation**	**ICC**	**Results of evaluation**	**AUC**	**Sensitivity**	**Specificity**	**Results of evaluation**	**Indicators**	**Results of evaluation**
TICS-M [34]	NA	NA		D	NA	NA		NA	0.61	0.48	0.7	V	2	V
ACE-R [35]	D	D		NA	0.88	V		NA	0.93	0.89	0.83	V	2	V
ACE-III-S [36]	NA	D	CFI 0.67	V	NA		NA		NA					D
AV-MoCA [37]	D	D	CFI 0.988	V	0.76	V		NA	0.85			V	1	V
HKBC [38]	D	D		D	0.79	V		I	0.96	0.90	0.86	V	1	V
MoCA-S [59]	NA	D		NA	0.77	V		D	0.90			V	1	V
RCS – TR [40]	D	D		D	0.86	V	0.80	V	0.72			V		NA
MoCA -P [41]	NA	NA		NA	0.90	V		NA	0.85	0.81	0.77	V	1	V
MoCA – J [42]	D	D		NA	0.74	V	0.88	I	0.95	0.93	0.89	V	2	V
TorC-iPad version [43]	D	D		D	0.73	V		D	0.84	0.92	0.91	V	The correlation coefficient with the gold standard is 0.64.	V
SCEB-I [44]	D	D		D	NA	NA		NA	0.8	0.70	0.875	V	3	V
COMPASS [45]	D	D		D	NA	NA		NA	0.83	0.92	0.52	V	5	D
K-mMMSE [46]	D	D		D	0.91	V		I	0.92	0.86	0.79	V	1	V
RCS-T [47]	NA	D		D	0.71	V		NA	0.86	0.74	0.88	V	2	V
MoCA-K [48]	D	D		NA	0.86	V	0.75	V	0.94	0.89	0.84	V	2	V
Qmci-TW [19]	NA	D		NA	0.85	V	0.87	V	0.89			V	2	V
Qmci-G [49]	NA	D		D	0.71	V		NA	0.96	0.92	0.91	V	2	V
MoCA – BR [50]	D	D		NA	0.75	V	0.76	A	0.80	0.81	0.77	V	2	V
Qmci-J [51]	NA	D		NA	NA	NA		NA	0.74	0.94	0.72	V	1	D
TYM-S [52]	D	D		D	0.78	V		NA	0.96	0.86	0.69	V	4	V
MoCA [53]	D	D		NA	0.83	V		NA				NA	1	V
ACE-III [54]	NA	NA		NA	NA	NA			0.85	0.92	0.63	V		NA
M@T [55]	D	D	NA	NA	0.92	V		D	0.93	0.96	0.79	V	1	V
MoCA – BM [56]	D	D		NA	0.80	V		D	0.82	0.90	0.87	V	1	V
PGPCOG [57]	D	D		D	0.978			D						
MoCA – J [58]	NA	NA	CFI 0.97	V	0.89	V		NA	NA					NA
MoCA-S [39]	NA	NA		NA	0.64	D		NA	0.79	0.77	0.74	V	1	V
TUA-WELLNESS [60]	D	D		D	NA	NA		D	0.84	0.83	0.73	V	6	V
SIS [61]	NA	D		NA	0.70	V		NA	0.93	0.86	0.87	V	1	V
MocA-BJ [62]	NA	D		NA	0.88	V		D	0.71	0.69	0.64	V	1	V
ACE-III-C [63]	NA	NA		NA	0.83	V	0.81	V	0.90	0.96	0.82	V	2	V

*Abbreviations:* *A *Adequate, *D *Doubtful, *I *Inadequate, *NA *Not applicable, *AUC *The area under the curve.

Regarding instrument development, none of the instruments reported a theoretical model, and the absence of a description detailing the quality control of the instrument development process, such as the conduct of cognitive interviews or pre-tests, resulted in a methodological quality that was characterized as"doubtful". Regarding the evaluation of content validity assessment, six studies lacked content validity measures, and the remaining studies were rated as"doubtful"due to their failure to concurrently assess comprehensibility and comprehensiveness. The methodological quality was therefore deemed"doubtful". The aforementioned studies did not employ adequate sample sizes, failed to clarify whether a group meeting was conducted, and did not ensure the accuracy of the interview transcriptions.

The internal structure of the instruments was evaluated in terms of structural validity, internal consistency, and cross-cultural validity/measurement invariance [66]. However, only structural validity and internal consistency were measured in the included studies.

Only three studies [36, 37, 58] provided specific statistical values, employing Confirmatory Factor Analysis (CFA) and Exploratory Factor Analysis (EFA), as recommended by the COSMIN guidelines. A study [37] with a sample size of 114 cases (meeting the criterion that the sample size should be 7 times the number of entries or ≥ 100 cases) reported an RMSEA (90% *CI*) of 0.067 (0.000- 0.139), CFI > 0.90, indicating an acceptable fit. Another study [18] conducted CFA and EFA with a sample size of 1204, performing CFA for each dimension of the scale, and reporting all CFI values > 0.90, indicative of a good fit. As none of the three studies identified any other methodological flaws, the methodological quality was rated as"very good". Eleven studies received a"doubtful"rating due to concerns regarding the applicability of the chosen model to the study and the inadequate sample size. Besides, the content validity of the scale was assessed by calculating the correlation coefficients between the scores of the items in the scale and the total score of the items in the studies included in this study. However, a study [53] did not adhere to reactivity modeling, which, according to the COSMIN risk of bias evaluation checklist [29], warrants its exclusion from consideration. The internal consistency of each unidimensional scale was calculated for 23 studies, resulting in an overall methodological quality rating of"very good". However, the study by Špeh et al. (2024) [59] solely calculated item-overall correlations, resulting in an assessment of methodological quality that was deemed"doubtful".

In terms of reliability, four studies [19, 40, 48, 63] calculated the Intraclass correlation coefficients (ICC) describing the ICC models/formulas, and therefore, the methodological quality was rated"very good". One study [50] calculated the ICC without providing a detailed description of the formula, resulting in a methodological quality rating of"adequate". The COSMIN guidelines emphasize that the consistency of the time interval between the two measurements, the subjects of the measurements, and the measurement setting are crucial indicators for assessing the test–retest reliability of an instrument [64]. Seven studies calculated only Pearson or Spearman coefficients and did not describe the time interval, resulting in a methodological quality rating of"doubtful". Three studies [38, 42, 46] reported inappropriate time intervals, leading to a methodological quality rating of"inadequate".

Regarding the validity of the studies, the area under the curve (AUC), sensitivity, and specificity were calculated for all 27 studies, resulting in an overall methodological quality rating of"very good".

In the context of hypothesis testing, a total of 24 studies were examined. These studies were evaluated in conjunction with a meticulously designed control instrument, resulting in an overall methodological quality rating of"very good". However, three studies [36, 45, 51] exhibited minor methodological deficiencies, leading to an assessment of methodological quality rating as"doubtful".

### Psychometric properties of instruments and quality of evidence

According to the COSMIN guidelines [31], reviewer ratings determine content validity in the absence of content validity studies and when the risk of bias for PROM development studies is rated as"inadequate". The quality of content validity in 22 studies was rated as “inconsistent”. The quality of content validity in studies such as Memõria et al. (2013) was rated as “inconsistent”, and the quality of the evidence was reduced by one level due to the"doubtful"rating of the risk of bias for content validity, to “moderate quality”. Five studies experienced a downgrade of three evidence levels according to the COSMIN modified evidence grading method because they did not report content validity. Three studies [37, 38, 49] examined psychometric properties that were rated as"adequate". Table 4 demonstrates the results of the quality of evidence ratings of the included studies.

**Table 4:** Summary of results for psychometric properties and evidence

Patient-reported outcome measure	Content validity			Structural validity			Internal consistency			Reliability			Criterion validity			Hypothesis testing for construct validity		Recommendation
	**QM**	**QE**		**QM**	**QE**		**QM**	**QE**		**QM**	**QE**		**QE**	**QM**		**QE**	**QM**	
TICS-M [34]	NA	Very low			Moderate		NA	NA		NA	NA		-	High		?	High	C
ACE-R [35]	±	Moderate		NA	NA		?	NA		NA	NA		+	Moderate		?	Moderate	B
ACE-III-S [36]	±	Moderate		+	High		NA	NA		NA	NA			NA		+	Moderate	B
AV-MoCA [37]	+	Moderate		+	High		+	High		NA	NA		+	High		?	NA	A
HKBC [38]	+	Moderate		?	Moderate		+	Moderate		?	Low		+	High		?	High	A
MoCA-S [59]	±	Moderate		NA	NA		?	NA		?	Low		+	1Moderate		NA	Moderate	B
RCS – TR [40]	NA	Moderate		?	Moderate		+	very low		+	Low		+	Low		NA	NA	B
MoCA -P [41]		Very low		NA	NA		?	NA		NA	NA		+	Moderate		?	Moderate	B
MoCA – J [42, 58]	±	Moderate		+	High		+	High		+	Low		±	High		?	High	B
TorC-iPad version	±	Moderate		?	Moderate		+	Moderate		?	Low		+	Moderate		?	Moderate	B
SCEB-I	±	Moderate			Moderate		NA	NA		NA	NA		+	Low		?	Low	B
COMPASS [45]	±	Moderate		?	Moderate		NA	NA		NA	NA		+	Moderate		?	Moderate	B
K-mMMSE [46]	±	Moderate		?	Moderate		+	Moderate		?	Very low		+	Moderate		?	Moderate	B
RCS-T [47]	±	Moderate		?	Moderate		+	Low		NA	NA		+	Moderate		?	Moderate	B
MoCA-K [48]	±	Moderate		NA	NA		?	NA		+	Low		+	Low		?	Low	B
Qmci-TW [19]	±	Moderate		NA	NA		?	NA		+	Low		+	Low		?	Low	B
Qmci-G [49]	+	Moderate		?	Moderate		+	Low		NA	NA		+	Moderate		?	Moderate	A
MoCA – BR [50]	NA	Moderate		NA	NA		?	NA		+	Moderate		+	Moderate		?	Moderate	B
Qmci-J [51]	±	Moderate		NA	NA		NA	NA		NA	NA		+	High		?	Moderate	B
TYM-S [52]	±	Moderate		?	Moderate		+	Moderate		NA	NA		+	Low		?	Low	B
MoCA [53]	±	Moderate		NA	NA		?	NA		NA	NA		NA	NA		?	Moderate	B
ACE-III [54]	NA	Very low		NA	NA		?	NA		NA	NA		+	High		NA	NA	B
M@T [55]	±	Moderate			Moderate		+	Moderate		+	Very low		+	Moderate		?	High	B
MoCA – BM [56]	NA	Very low		NA	NA		?	NA		?	Moderate		+	High		?	High	C
PGPCOG [57]	±	Moderate		?	Moderate		+	High		?	Moderate		NA	NA		NA	NA	B
MoCA-S [39]	NA	Very low		NA	NA		?			NA	NA		?	Moderate		?	Moderate	B
TUA-WELLNESS [60]	±	Moderate		?	Moderate		NA	NA		?	Moderate		+	High		?	High	B
SIS [61]	NA	Very low		?	Moderate		+	Moderate		NA	NA		+	High		?	High	B
MocA-BJ [62]	±	Moderate		NA	NA		?	NA		NA	Moderate		+	High		?	High	B
ACE-III-C [63]	NA	Very low		NA	NA		+	High		+	Moderate		+	Moderate		?	Moderate	B

(1) Overall rating rated as Sufficient (+), Intermediate (?), Insufficient (−), Inconsistent (±). (2) QE, quality of evidence; QM, quality of measurement. (3) NA stands for"Not Applicable."

In terms of structural validity, the psychological attributes in three studies ([36, 37, 58]) were rated as"adequate", and the quality of the evidence was determined to be"high quality". However, a total of fourteen studies did not report information regarding the"sufficient"symbol. Consequently, the psychometric properties of these studies were rated as"indeterminate", leading to a reduction in the level of evidence by one tier due to concerns regarding risk of bias.

The psychometric properties of four studies ([36, 37, 57, 58]) were determined to be"adequate"based on the calculation of Cronbach's alpha coefficients, which were found to be 0.7 or greater. This indicated that the quality of the evidence remained consistent and did not undergo a reduction. Accordingly, the psychometric properties were all"adequate", and the quality of evidence was not reduced. However, eleven studies lacked structural validity and did not meet the criteria for low-level evidence rating necessary to support structural validity. These studies were not subject to an evaluation of quality evidence. The quality of structural validity serves as the foundation for assessing the quality of evidence concerning internal consistency [64]. Consequently, five studies employed structural validity as the initial point of departure for evidence evaluation, resulting in equivalent quality of evidence ratings to the instrument's structural validity evidence ratings. Conversely, studies with samples of less than 100 cases, such as Manser and de Bruin (2024) and Koc Okudur et al. (2019), experienced a one-level reduction in the level of evidence due to imprecision. Similarly, a study [40] with a sample size of less than 50 cases, experienced a two-level reduction due to imprecision.

Three studies [36, 37, 58] exhibited ICC ranging from 0.75 to 0.88, with all values exceeding 0.7, thereby classifying the psychometric properties as"adequate". The remaining studies did not disclose ICC values, consequently leading to an assessment of the psychometric properties as"indeterminate". Six studies experienced a one-level downgrade in quality of evidence due to imprecision or risk of bias, and seven studies experienced a two-level downgrade due to imprecision and risk of bias.

One study [34] exhibited an AUC of 0.61, falling short of the 0.7 threshold. Consequently, its psychometric properties were evaluated as"insufficient". Conversely, the remaining studies demonstrated AUC values ranging from 0.71 to 0.96, surpassing the 0.7 benchmark. As a result, their psychometric properties were classified as"adequate". Eleven studies experienced a one-level downgrade in quality of evidence due to risk of bias or imprecision, and five studies experienced a two-level downgrade due to very serious imprecision or very serious risk of bias.

The panel found that the results of Calderón et al. (2021) were consistent with the hypothesis and therefore the psychometric properties were"adequate". The remaining studies lacked a clear hypothesis and were therefore rated as"indeterminate". Fourteen studies experienced a one-level downgrade in quality of evidence due to risk of bias or imprecision. Notably, two studies [19, 44] experienced a two-level downgrade due to severe imprecision. Similarly, Muñoz-Neira et al. (2014) observed a two-level reduction in the quality of evidence level, attributed to a significant risk of bias. Conversely, the remaining studies did not undergo a reduction in the level of evidence hierarchy.

### Recommendation levels of tools

The recommendation levels of the 30 tools are shown in Table 3. According to the COSMIN guidelines, the content validity and internal consistency of AV-MoCA, HKBC, and Qmci-G [37, 38, 49] were deemed"adequate", with a level of evidence of at least"moderate", constituting a Level A recommendation. The TICS-M [34] study, however, exhibited inadequate psychometric properties, thus resulting in a Class C recommendation and exclusion from the recommendation. The remaining 26 instruments were all recommended within Category B and have potential for application; however, further research is needed to assess their psychometric properties.

## Discussion

This systematic review involved an extensive search across eight databases, followed by literature screening, ultimately yielding a total of 31 articles. The risk of methodological bias, psychometric properties, ratings, and quality of evidence of 30 different assessment tools for screening older patients for MCI in primary care settings were assessed according to the COSMIN methodology. The results of this study may provide practical tips for future research and evidence-based guidance for researchers and healthcare professionals when selecting patient-reported outcome measures to screen older adults for MCI prevalence.

### Methodological quality of screening MCI tools needs to be improved

The COSMIN guidelines emphasize that content validity is the most important measurement attribute of an instrument [64]. The methodological quality of the content validity of the tools included in this study was"doubtful", exhibiting very low to moderate quality of evidence. When assessing content validity, it is important for patients and professionals to be aware of the relevance, comprehensiveness, and understandability of the tool [64]. However, most studies have only relied on expert opinion to determine the relevance of entries and have not conducted cognitive interviews or qualitative research with older patients and their caregivers, which may have led to an inadequate assessment of the comprehensiveness and relevance of entries. In addition, most studies used quantitative research methods to assess content validity, which may make it challenging to adequately capture the semantic and cultural context of the items in a questionnaire or measurement tool [67]. Therefore, future studies should incorporate qualitative research methods, such as cognitive interviews or focus group discussions, to gain a deeper understanding of respondents'interpretations and responses to questionnaire items, which could enable researchers to identify and correct potential problems within the questionnaire with a more comprehensive perspective and to improve content validity [68, 69].

Structural validity is a core validation dimension in the theoretical conceptualization of scales [70]; however, only three of the included studies [36, 37, 58] used CFA or item response theory (IRT), as recommended by the COSMIN guidelines, to assess construct validity. Therefore, researchers should prioritize CFA to assess scale structure and report indicators of model fit. Furthermore, the inappropriate establishment of a"gold standard"for scales confounds hypothesis testing with validity criteria. According to the COSMIN guidelines, there is no generally definitive gold standard for patient-reported outcome measurement instruments, except when an original scale is simplified, in which case the original scale can be used as the gold standard for a newly developed short scale [64]. Except for one study [43], all studies used the MMSE or other cognitive scales as the gold standard, deviating from the COSMIN guidelines. In future studies, researchers should avoid equating widely used scales with a gold standard. Regarding hypothesis testing, none of the studies formulated a clear hypothesis. Researchers should develop a clear hypothesis to improve the reliability and validity of the results.

In the context of reliability, Cronbach's alpha(s) emerges as the sole reliability coefficient that can be derived from a single-application scale [71]. This coefficient has been employed by most studies to assess internal consistency [44, 71]. However, in five studies, researchers did not assess internal consistency. Failure to assess internal consistency can lead to inaccurate estimates of reliability. Therefore, researchers must prioritize internal consistency and calculate it for unidimensional scales or subscales. Regarding reliability, only four studies reported ICCs and did not explicitly control for conditions such as measurement intervals and environmental stability, assessor training, etc. Therefore, researchers must synchronize the assessment of intertemporal stability and cross-assessor consistency of scales.

In addition, there was a significant paucity of evidence on cross-cultural validity and measurement error, and the responsiveness of current screening tools for older adults with MCI. To enhance the methodological quality of screening tools for older adults with MCI, it is essential to implement the COSMIN guidelines in a systematic manner. This will ensure the integrity and comprehensiveness of the study design and analysis process.

### Recommendation of the screening tools for MCI

This study systematically evaluated 30 MCI screening tools based on the COSMIN guidelines. Three tools, AV-MoCA [37], HKBC [38], and Qmci-G [49], demonstrated superior psychometric properties among the measures examined.

The AV-MoCA represents a telemedicine-adapted cognitive screening tool designed for older adults with limited mobility or in remote areas, and its audio-visual interaction model ensures the quality of the screening while significantly reducing the cost of implementation [37]. The HKBC is tailored to Asian cultures, featuring a brief 7-min administration protocol. Employing low-complexity inputs can effectively mitigate the bias related to educational levels. As a result, the HKBC is especially suitable for populations with low educational attainment [38]. The Qmci-G addresses the time constraints of primary care settings with an ultra-short 3 ~ 5 min time frame, enabling efficient initial screening and subsequent triage while optimizing healthcare resource allocation [49]. However, the cross-cultural applicability of the above tools still needs to be carefully verified, and their input may affect the accuracy of screening due to factors such as semantic understanding and task familiarity caused by national/regional cultural differences. TICS-M [34] is classified as category C due to insufficient reliability, which carries a high risk of misdiagnosis, and is not recommended for clinical use. The remaining 26 Category B tools need to be combined with a multidimensional assessment strategy: their reliability performance should be dynamically monitored in use and combined with neuroimaging, biomarkers, or functional assessment to synthesize the results. Future practice could explore the combined application model of Class A and Class B tools, such as combining AV-MoCA remote review after initial screening with Qmci-G to balance efficiency and accuracy.

### Implications for future practice and research

The utilization of an MCI screening instrument within the geriatric population has been demonstrated to promote the early detection and diagnosis of mild MCI, thus facilitating the provision of timely intervention and support for patients [14]. The findings of this study offer researchers a foundation upon which to select a high-quality MCI assessment tool that is most suitable for older adults in the region. This selection could enhance the accuracy of assessment results and facilitate the early and accurate diagnosis of MCI, consequently improving patient prognosis. Furthermore, the results of this study provide scientific recommendations for the continuous improvement of existing assessment tools.

In the realm of research, linguistic differences and cultural values have been shown to significantly influence the expression of cognitive functions [72]. Consequently, future research endeavors should prioritize the cross-cultural adaptation of the tool to ensure its applicability in diverse cultural contexts. This should entail the development and validation of multilingual versions [73]. Besides, there is a need for methodological optimization to enhance the content validity of the tool through a mixed study design, and to improve the construct validity and measurement accuracy by using a joint validation strategy of CFA and IRT (Item Response Theory). Moreover, with the development of digital health technologies, future studies should explore the possibility of integrating MCI screening tools with digital platforms to improve the convenience and accessibility of screening.

### Limitations

First, the study's inclusion criteria limited the sample to English and Chinese publications, thereby excluding potentially relevant studies in other languages. This approach potentially introduced selection bias, as key studies might have been overlooked, thereby affecting the comprehensiveness of the analysis. Secondly, the study employed a dual approach of manual searching and electronic database querying. While intended to broaden the range of studies, this approach introduced elements of subjective judgment during the initial selection and detailed review stages. Despite a collaborative approach to reconcile academic differences, there was a risk of interpretation bias in the selection and analysis of studies. Finally, the psychometric properties of most instruments were assessed by only a single study, which may affect the reliability of the findings.

## Conclusion

The present systematic review identified and described 30 instruments and their psychometric properties. The findings suggest that the AV-MoCA, HKBC, and Qmci-G instruments can assess MCI in older adults. It is recommended that the aforementioned research tools be used to screen older adults for MCI, although emphasis should be placed on cultural and regional validity. The results of the included studies emphasize the necessity for future research to develop or translate more assessment tools suitable for screening for MCI in older adults with appropriate characteristics. Furthermore, our results emphasize the importance of adopting more rigorous standards when evaluating and reporting the psychometric properties of assessment instruments in accordance with the COSMIN guidelines.

## Supplementary Information

Supplementary Material 1.

## Data Availability

The author confirms that all data generated or analyzed during this study are included in this published article.
