# Supplementary material for: Psychometric properties of screening tools for mild cognitive impairment in older adults based on COSMIN guidelines: a systematic review
Source: BMC Geriatr. 2025 Jun 2;25:401. doi: 10.1186/s12877-025-06030-4 (PMC12128237; doi:10.1186/s12877-025-06030-4)
Supplement: Supplementary file 1 — Supplementary Material 1. [file 12877_2025_6030_MOESM1_ESM.docx]

**Supplementary File 1: Search strategies of each database**

**PubMed**

| **Search** | **Query** |
| --- | --- |
| #1 | cognitive dysfunction[MeSH Terms] |
| #2 | ((((((cognitive dysfunction*[Title/Abstract]) OR (mild cognitive impairment*[Title/Abstract])) OR (cognitive disorder*[Title/Abstract])) OR (cognitive decline*[Title/Abstract])) OR (mental deterioration*[Title/Abstract])) OR (mild cognitive disorder*[Title/Abstract])) OR (MCI[Title/Abstract]) |
| #3 | (scale*[Title/Abstract] OR questionnaire*[Title/Abstract] OR survey*[Title/Abstract] OR checklist*[Title/Abstract] OR tool*[Title/Abstract] OR measure*[Title/Abstract] OR assessment[Title/Abstract]) OR ("Surveys and Questionnaires"[Mesh]) OR ("Patient Reported Outcome Measures"[Mesh]) |
| #4 | (screen[Title/Abstract]) OR (screening[Title/Abstract]) |
| #5 | #1 AND #2 AND #3 AND #4  Items found: 1697 |

**EMBASE**

| **Search** | **Query** |
| --- | --- |
| #1 | ((((((cognitive dysfunction*:ab,ti) OR (mild cognitive impairment*:ab,ti)) OR (cognitive disorder*:ab,ti)) OR (cognitive decline*:ab,ti)) OR (mental deterioration*:ab,ti)) OR (mild cognitive disorder*:ab,ti)) OR (MCI:ab,ti) |
| #2 | 'screen':ti OR 'screening':ti |
| #3 | scale*:ab,ti OR questionnaire*:ab,ti OR survey*:ab,ti OR checklist*:ab,ti OR tool*:ab,ti OR measure*:ab,ti OR assessment:ab,ti |
| #4 | (psychometr* OR 'outcome assessment' OR 'observer variation' OR reproducib* OR reliab* OR unreliab* OR valid* OR 'coefficient of variation' OR coefficient OR homogeneity OR homogeneous OR 'internal consistency' OR test-retest OR stability OR interrater OR inter-rater OR intrarater OR intra-rater OR intertester OR inter-tester OR intratester OR intra-tester OR interobserver OR inter-observer OR intraobserver OR intra-observer OR intertechnician OR inter-technician OR intratechnician OR intra-technician OR interexaminer OR inter-examiner OR intraexaminer OR intra-examiner OR interassay OR inter-assay OR intraassay OR intra-assay OR interindividual OR inter-individual OR intraindividual OR intra-individual OR interparticipant OR inter-participant OR intraparticipant OR intra-participant OR kappa* OR generaliza* OR generalisa* OR concordance OR discriminative OR 'known group' OR 'factor analysis' OR 'factor analyses' OR 'factor structure' OR 'factor structures' OR dimension* OR subscale* OR 'item discriminant' OR 'interscale correlation*' OR error OR errors OR 'individual variability' OR 'interval variability' OR 'rate variability' OR 'standard error of measurement' OR sensitiv* OR responsive* OR 'minimal detectable concentration' OR interpretab* OR 'meaningful change' OR 'ceiling effect' OR 'floor effect' OR 'Item response model' OR IRT OR Rasch OR 'Differential item functioning' OR DIF OR 'computer adaptive testing' OR 'item bank' OR 'cross-cultural equivalence'):ab,ti |
| #5 | #1 AND #2AND #3 AND #4  Items found: 61 |

**Web of Science （MEDLINE）**

| **Search** | **Query** |
| --- | --- |
| #1 | TI=(cognitive dysfunction* OR mild cognitive impairment* OR cognitive disorder* OR cognitive decline* OR mental deterioration* OR mild cognitive disorder* OR MCI) |
| #2 | TS=(scale* OR questionnaire* OR survey* OR checklist* OR tool* OR measure* OR assessment OR patient reported outcome measures ) |
| #3 | TS=(screen OR screening) |
| #4 | ((((((((((((AB=(psychometr* OR outcome assessment OR observer variation OR reproducib* OR reliab* OR unreliab* OR valid* OR coefficient of variation OR coefficient OR homogeneity OR homogeneous OR internal consistency OR test-retest OR stability OR interrater OR inter-rater OR intrarater OR intra-rater OR intertester OR inter-tester OR intratester OR intra-tester OR interobserver OR inter-observer OR intraobserver OR intra-observer OR intertechnician OR inter-technician OR intratechnician OR intra-technician OR interexaminer OR inter-examiner OR intraexaminer OR intra-examiner OR interassay OR inter-assay OR intraassay OR intra-assay OR interindividual OR inter-individual OR intraindividual OR intra-individual OR interparticipant OR inter-participant OR intraparticipant OR intra-participant OR kappa* OR generaliza* OR generalisa* OR concordance OR discriminative OR known group OR factor analysis OR factor analyses OR factor structure OR factor structures OR dimension* OR subscale* OR item discriminant OR interscale correlation* OR error OR errors OR individual variability OR interval variability OR rate variability OR standard error of measurement OR sensitiv* OR responsive* OR minimal detectable concentration OR interpretab* OR meaningful change OR ceiling effect OR floor effect OR Item response model OR IRT OR Rasch OR Differential item functioning OR DIF OR computer adaptive testing OR item bank OR cross-cultural equivalence)) OR TS=(instrumentation or methods or psychometrics or Outcome Assessment, Health Care or observer variation or Health Status Indicators or reproducibility of results or discriminant analysis)) OR AB=(cronbach* and (alpha or alphas))) OR AB=(item and (correlation* or selection* or reduction*))) OR AB=(test and retest)) OR AB=(reliab* and (test or retest))) OR AB=(intraclass and correlation*)) OR AB=(multitrait and scaling and (analysis or analyses))) OR AB=(variability and (analysis or values))) OR AB=(uncertainty and (measurement or measuring))) OR AB=(limit and detection)) OR AB=((minimal or minimally or clinical or clinically) and (important or significant or detectable) and (change or difference))) OR AB=(small* and (real or detectable) and (change or difference)) |
| #5 | #1 AND #2 AND #3 AND #4  Items found: 2843 |
| #5 | #1 AND #2 AND #3 AND #4  Items found: 2843 |

**Scopus**

| **Search** | **Query** |
| --- | --- |
| #1 | TITLE-ABS-KEY(cognitive dysfunction* OR mild cognitive impairment* OR cognitive disorder* OR cognitive decline* OR mental deterioration* OR mild cognitive disorder* OR MCI) |
| #2 | TITLE-ABS-KEY(screen OR screening) |
| #3 | TITLE-ABS-KEY(scale* OR questionnaire* OR survey* OR checklist* OR tool* OR measure* OR assessment OR patient reported outcome measures) |
| #4 | #1 AND #2 AND #3 AND #4  Items found: 26 |

**CNKI**

| **Search** | **Query** |
| --- | --- |
| #1 | SU%=('轻度认知障碍') |
| #2 | FT%=('筛查') |
| #3 | FT%=('问卷'+'量表'+'工具') |
| #4 | FT%=('信度'+'效度'+'跨文化'+'测量学特性'+'测量属性') |
| #5 | #1 AND #2 AND #3 AND #4  Items found: 466 |

**WanFang**

| **Search** | **Query** |
| --- | --- |
| #1 | 全部:(轻度认知障碍) |
| #2 | 全部:(筛查) |
| #3 | 全部:(问卷 or 量表 or 工具) |
| #4 | 全部:(信度 or 效度 or 跨文化 or 测量学特性 or 测量属性) |
| #5 | #1 AND #2 AND #3 AND #4  Items found: 101 |

全部文献数量：4509＋567=5076

其他渠道：0

去重后：

Records screened by title and abstract：1793

Full-text articles assessed for eligibility：154

Language ineligibility (n =)

-Population ineligibility (n = )

-No measurement properties (n = )

-Type of study ineligible (n = )

-Repeat publication (n = )

-Full text not available (n = )
